# Inbreeding Avoidance Influences the Viability of Reintroduced Populations of African Wild Dogs (*Lycaon pictus*)

**DOI:** 10.1371/journal.pone.0037181

**Published:** 2012-05-16

**Authors:** Penny A. Becker, Philip S. Miller, Micaela Szykman Gunther, Michael J. Somers, David E. Wildt, Jesús E. Maldonado

**Affiliations:** 1 Center for Conservation and Evolutionary Genetics, Smithsonian Conservation Biology Institute, National Zoological Park, Smithsonian Institution, Washington, District of Columbia, United States of America; 2 Center for Species Survival, Smithsonian Conservation Biology Institute, National Zoological Park, Smithsonian Institution, Front Royal, Virginia, United States of America; 3 Centre for Wildlife Management, University of Pretoria, Pretoria, South Africa; 4 Conservation Breeding Specialist Group (Species Survival Commission/International Union for Conservation of Nature), Apple Valley, Minnesota, United States of America; 5 Department of Wildlife, Humboldt State University, Arcata, California, United States of America; 6 Centre for Invasion Biology, University of Pretoria, Pretoria, South Africa; 7 Department of Vertebrate Zoology, National Museum of Natural History, Smithsonian Institution, Washington, District of Columbia, United States of America; University of Western Ontario, Canada

## Abstract

The conservation of many fragmented and small populations of endangered African wild dogs (*Lycaon pictus*) relies on understanding the natural processes affecting genetic diversity, demographics, and future viability. We used extensive behavioural, life-history, and genetic data from reintroduced African wild dogs in South Africa to (1) test for inbreeding avoidance via mate selection and (2) model the potential consequences of avoidance on population persistence. Results suggested that wild dogs avoided mating with kin. Inbreeding was rare in natal packs, after reproductive vacancies, and between sibling cohorts (observed on 0.8%, 12.5%, and 3.8% of occasions, respectively). Only one of the six (16.7%) breeding pairs confirmed as third-order (or closer) kin consisted of animals that were familiar with each other, while no other paired individuals had any prior association. Computer-simulated populations allowed to experience inbreeding had only a 1.6% probability of extinction within 100 years, whereas all populations avoiding incestuous matings became extinct due to the absence of unrelated mates. Populations that avoided mating with first-order relatives became extinct after 63 years compared with persistence of 37 and 19 years for those also prevented from second-order and third-order matings, respectively. Although stronger inbreeding avoidance maintains significantly more genetic variation, our results demonstrate the potentially severe demographic impacts of reduced numbers of suitable mates on the future viability of small, isolated wild dog populations. The rapid rate of population decline suggests that extinction may occur before inbreeding depression is observed.

## Introduction

Mating with kin has been shown to lead to decreased heterozygosity, expression of deleterious alleles, and reduced fitness due to inbreeding depression in a variety of species [1], [2]. Although the short-term effects can be morphological abnormalities [3], decreased reproductive success [4], and greater susceptibility to disease [5], the long-term consequences can be reduced ability to adapt to environmental change [6] and an increased risk of extinction [7]. As a result, natural selection should favour behavioural mechanisms for animals to avoid mating with kin, particularly in species that could potentially suffer the most severe costs of inbreeding depression [8]. There are three recognized behavioural strategies associated with inbreeding avoidance. The first is that natal dispersal reduces contact among relatives, an approach commonly found in species like the black-tailed prairie dog (*Cynomys ludovicianus*) that displays male-biased dispersal and female philopatry [9]. In the second, females seek extra-pair matings to enhance the genetic diversity of progeny, as observed in the blue tit (*Parus caeruleus*) [10]. In the third, individuals avoid mating with relatives via three types of kin recognition: 1) familiarity (e.g., Seychelles warbler, *Acrocephalus sechellensis*) [11]; 2) major histocompatibility complex comparisons (e.g., house mouse, *Mus musculus*) [12]; or 3) phenotype matching, where an individual compares templates of close kin or itself to determine relatedness to unknown individuals (e.g., golden hamster, *Mesocricetus auratus*) [13].

Endangered species [14], cooperative breeders with high reproductive skew towards selected individuals [15], and small, reintroduced populations [16], [17] are particularly vulnerable to losses of genetic diversity and inbreeding depression. Although the consequences of homozygosity are well known, little attention has been directed at the specific behaviours used to avoid inbreeding. No doubt this is due, in part, to the need for a comprehensive, longitudinal database on the life history, genetics, and breeding behaviour of targeted species. It also is possible that these behaviours are not prevalent in some species because the cost of avoiding mating between relatives outweighs the genetic consequences arising from inbreeding depression [18]. As inbreeding avoidance further restricts numbers of suitable mates available to reproduce, avoidance costs could be quite high for species living in low density, fragmented populations and for those with mating systems involving few breeders [19]–[21]. However, it is not yet known whether endangered species (which generally are undergoing both significant habitat loss and population declines) rely on mechanisms of inbreeding avoidance, or if these behaviours affect future viability.

To improve our understanding of inbreeding avoidance and its consequences, we examined both real and simulated data based on a reintroduced population of African wild dogs (*Lycaon pictus*) in KwaZulu-Natal province (KZN), South Africa. Although some larger-sized wild dog populations have remained intact in the historical range, many (including those in South Africa) are relatively small due to limited availability of continuous habitat. Previous studies by others have indicated that inbreeding with first-order relatives (parents, offspring, and siblings) may be rare in this species due to long-distance and, in some regions, sex-biased dispersal [22], [23]. However, wild dogs are cooperative breeders living in highly social groups with mature offspring often remaining in the natal pack to help raise pups for 1 to 3 years before dispersal [23]. The primary factor generally believed to regulate reproductive success (and inbreeding avoidance) is behavioural dominance displayed by the alpha male and female who, in turn, behaviourally and/or physiologically suppress reproduction in remaining pack members [24].

However, if only dominance prevents offspring from breeding in the natal pack, then at least three other outcomes would be common. First, when the dam or sire dies or disappears, an offspring would breed with the opposite sex parent. Second, siblings would breed together after dispersal from the natal pack. Third, offspring, siblings, and adults unrelated to the alpha pair should be equally suppressed from reproducing in the natal pack. However, this third supposition has been rejected as we recently presented evidence of significant reproductive sharing in this species, whereby brothers of the dominant male and sisters of the dominant female participate in breeding [25]. Another point of relevance is derived from the earliest efforts at reintroducing wild dogs to KZN. After release of the founders in 1980 and 1981 that led to the formation of a single pack, reproduction stopped in 1987 through 1989 and again from 1994 through 1996. By 1996 only five individuals remained in the population [26], [27], and it was speculated that reproduction may have ceased because only close relatives remained (although not confirmed with genetic or pedigree data) [26].

The present study had two aims. The first was to examine the possibility that African wild dogs avoid inbreeding through selective mating. The second was to explore the persistence of this species, given its dire status, naturally low densities, and often small population sizes. These factors plus the existence of strong inbreeding aversion inevitably will cause even more challenges for wild dogs to find unrelated mates. Thus, we explored through simulation modelling the extinction risk associated with different inbreeding thresholds.

Our first hypothesis was that African wild dogs avoid inbreeding beyond the restrictions of established dominance, and that some type of inherent kin recognition likely prevents matings between familiar relatives. We tested for behavioural inbreeding avoidance in wild dogs between: 1) parents and reproductively mature offspring in the natal pack, 2) parents and mature offspring after a reproductive vacancy, and 3) adult siblings at or after the time of dispersal. Using an extensive genetic database, we also evaluated the influence of relatedness on mate choice by comparing the relatedness of confirmed breeding pairs to the mean pairwise values of individuals with known relationships, as well as to pairs within the population that might have mated but did not. Our second hypothesis was that inbreeding avoidance had a significant negative impact on the reproductive potential of wild dogs, which would increase the likelihood of extinction of small, fragmented populations. We expected that simulation modelling would show these population-limiting effects given that earlier studies have demonstrated the demographic vulnerability of this species to extinction when pack sizes or numbers fall below a critical threshold due to deterministic or stochastic fluctuations [27]. This examination took advantage of a substantial database on wild dog population-specific demographic and behavioural data. Recently developed population viability analysis tools were used to examine the sensitivity of African wild dogs to different levels of inbreeding and future population trends.

## Methods

### Ethics Statement

This work was done with the permission and relevant permits from the local government authority, Ezemvelo KZN Wildlife, and was approved by the Smithsonian National Zoological Park IACUC protocol no 08-21 and Humboldt State University IACUC, protocol no. 06/07.W.209.A. Whenever possible, non-invasive sampling methods were utilized to collect genetic material. In addition, immobilization was conducted only for collaring or translocation purposes supervised by wildlife veterinarians and/or managers.

### Study Population

Intensive demographic and behavioural monitoring was conducted for the reintroduced African wild dog population in KZN province from August 1997 through December 2008. After initial multiple releases into Hluhluwe-iMfolozi Park (HiP) in the 1980s, total numbers of wild dogs dwindled to five adult individuals in a single, non-reproducing pack by 1996 [28], [29]. To stimulate population growth, additional packs were translocated to HiP in 1997 [30], 2001, and 2003 [31], [32] as well as to two other protected areas in the province in 2005 and 2006 [33]. Over this 11 year period, the collective population grew steadily through translocations, reproduction, dispersal, and new, natural group formations to nine breeding packs comprised of 88 total dogs in three protected areas. Our examination here focused on adult males and females that were alive and sexually mature from 1997 through 2008 (n = 207, including 111 males and 96 females). Our estimate of sexual maturity (>18 months old) was conservative given that we had occasionally observed that some males copulated at as young as 13 months and females conceived at 15 months. Of our total study population, 113 wild dogs (54.6%) from 10 packs were physically sampled for blood, tissue, and/or voided faeces to extract DNA to produce direct evidence of levels of genetic relatedness among individuals (see below).

### Demographic and Behavioural Data Collection

Data on pack composition (number of animals, age, and gender), life history information (births, dispersals, pack formations, deaths), dominance (hierarchy of individuals of each sex per pack), and reproductive status (mating, denning) were collected at least once and as often as 10 times per month. Details for these methods have been published in Spiering et al. [25], [34]. In short, individual wild dogs were identified by unique coat patterns and were individually known from birth or translocation to KZN. At least one and as many as four individuals per pack were fitted with VHF radio collars to facilitate the monitoring of packs from a vehicle or on foot.

Although reproductive sharing does occur in wild dogs, a majority of pups in the KZN population were produced by alpha males and females [25]. Therefore, behavioural determination of the dominant pair of each pack was used as an indicator that these individuals were mating together, but we also observed mating behaviour involving subordinates and resolved genetic parentage of pups when possible. The alpha male and female in a given pack were recognized on the basis of: 1) reciprocal male and female scent-marking behaviour [35], 2) obvious co-incidental male and female movement, and 3) mutual offensive and defensive manoeuvres in agonistic encounters with other adult pack members [23].

### Genetic Analyses

Rather than assuming familial relationships within these cooperative breeding groups strictly on the basis of observing behaviours, we combined our longitudinal behavioural observations with molecular genetic data to determine specific pack member interrelationships. Biomaterial sampling for genetic evaluations was conducted from January 2003 through January 2008 using a combination of invasive and non-invasive approaches. Wild dog tissue and blood samples were obtained opportunistically during immobilization operations for translocation and collaring and when a wild dog carcass was located [34]. Faecal samples were collected fresh from known individuals within 5 to 30 min of deposition and then stored in labelled, plastic freezer bags at −20°C until genetic analysis.

All individuals were genotyped at 17 dinucleotide microsatellite loci and two tetranucleotide loci that yielded 4.8 alleles per locus on average. These markers were consistent with other wild dog genetic studies and are commonly used for determining parentage in domestic dogs [25]. Specifics on DNA extraction, polymerase chain reaction (PCR) protocols, and methods used to detect and eliminate genotyping and sampling errors are discussed in Spiering et al. [25], [34]. Tests for deviation from Hardy-Weinberg equilibrium and tests for parentage in the present evaluation relied on the likelihood based approach in CERVUS software [36]. Locus INU030 was excluded from the parentage analyses because a significantly lower than expected frequency of heterozygotes was detected, indicating a high incidence of null alleles. No other locus deviated from Hardy-Weinberg equilibrium. The simulation program in CERVUS was used to establish the critical difference in natural logarithm of the likelihood ratio (LOD score) between the first and second most likely candidate parents (at >95% confidence). Only adults from within the pack with a given set of offspring were considered candidate parents because no extra-group copulations have been reported for this species (and analyses later confirmed that all parentage was assigned to pack members). We included genotypes for all genetically sampled individuals from the population to calculate pairwise relatedness estimates (r) with the program KINSHIP (version 1.3.1) [37] and used the observed r values to determine Wright's inbreeding coefficients (F) [38].

### Tests of Inbreeding Avoidance

For our evaluation, and based on the observed *r* values derived from our population allelic frequencies, we considered first-order relationships to be parent-offspring or full sibling pairs. Half sibling and aunt/uncle-niece/nephew pairs were second-order kin, and first cousin pairs were third-order relatives. Since all breeding occurred within established wild dog packs, we tested for inbreeding avoidance by determining the frequency of situations in which packs included breeding pairs that were related. Specifically, the number of situations in which inbreeding might have occurred (both individuals were alive, sexually mature, and in the same group) were compared to behavioural observations of mating between (1) parents and offspring in natal packs, (2) parents and offspring after reproductive vacancies, and (3) mature siblings after dispersal. Secondly, to determine if mating occurred between close kin, we compared the pairwise genetic relatedness of breeding pairs to the mean pairwise values of individuals with known relationships in our population. Lastly, as there is a relatively stable group structure within African wild dog packs (i.e., a separate social hierarchy for males and females with a dominant breeding pair) [39], we also assessed the influence of pairwise genetic relatedness on mate choice by comparing the relatedness in breeding pairs with pairs within the population that did not breed with each other.

### Statistical Analyses

We assessed the relationships between opportunities for inbreeding, observed incestuous matings, and population size by means of linear regression. A chi-squared test was used to compare observed and expected matings and mating opportunities between relatives and non-relatives. We compared pairwise relatedness values of dyads of known relationships, breeding pairs, and the entire population with Wilcoxon signed-rank tests. We used Student's t-tests to assess the maintenance of genetic diversity across varying inbreeding thresholds in our models. All statistical analyses were performed with JMP software version 3.2.2 (SAS Institute Incorporated), and means were given ± standard error of the mean, except where indicated.

### Population Viability Modelling

We used VORTEX (Version 9.95) [40], [41] population viability modelling software to evaluate the influence of inbreeding avoidance behaviours on population trends and extinction risk for the species. Each simulation was repeated 1,000 times and results predicted over 100 years. Demographic rates reported below include measures of annual environmental variation, expressed as standard deviations around the mean values of variables [41].

The existing extensive demographic database on the KZN African wild dog population was used for model development, with input from the published literature [39], [42], as appropriate. To mimic a realistic population demographic structure and pedigree, a studbook file that included all individuals alive in the KZN population in December 2008 was used as input to the model. To simulate the social and reproductive characteristics of African wild dogs within VORTEX, we used a combination of settings in the model to reflect accurate reproductive rates, including proportions of animals within packs and across the population that actually bred. First, to reflect that wild dog packs generally are relatively stable with the same groups of individuals mating over several years and the dominant individuals often breeding repeatedly [23], [39], ‘long-term monogamy’ was selected as the reproductive system. Although more reproductive sharing than previously expected was discovered in breeding packs, most pups were still produced by dominants (93% whelped by alpha females and 72% sired by alpha males) because many packs were comprised of only the alpha pair and their offspring [25]. Therefore, long-term monogamy captures the most important aspects of the wild dog reproduction scenario and is the closest approximation of the breeding system. We then calculated the percentages of males and females in the breeding pool, thereby dealing with the normative that only adult members of the pack have the opportunity to reproduce (i.e., excluding dispersing individuals or offspring remaining in natal packs). Thus, it was determined that an average of 48% of all adult males in the KZN population comprise the breeding pool, a value used throughout the analysis. For females, we added a function that would allow us to incorporate the percentage of individuals breeding based on specific age classes and whether or not a female had produced offspring in previous years. Only 7.7±3.0% (SD) of 2 year old females normally have reproduced because younger wild dogs are less likely to have already dispersed and joined a breeding pack. While only 29.7±10.0% of females >2 years old that had not whelped pups in previous years produce young, 95.6±10.0% of females that had already reproduced previously as the alpha or beta individual continue to breed, most often until death [25]. In KZN, African wild dog females whelp pups at 1.3 to 10 years old, and males sire offspring from 1.1 to 10 years old [25]. As most individuals did not breed before 2 years old, this was set as the age at first offspring production with maximum breeding age fixed at 10 years. Mean litter size in this population is 7.6±0.6 pups, with the largest litter recorded as 14 pups and near gender parity at this age class (0.51±0.04) [25]. Although VORTEX is known to be limited in ability to accurately portray the social complexities of some species [43], we were confident that our vast demographic and genetic background data allowed a robust PVA assessment. This was confirmed by discovering that: 1) the simulated population growth rate was comparable to the long-term, real-life data of the KZN population; and 2) the proportion of adult females breeding, adult female mortality, and disease were the most sensitive, important factors in the model, which was congruent with other wild dog PVA models [43], [44].

Using our demographic database, we calculated that the age-specific mortality rates in the KZN population were similar to those reported by Creel & Creel [39] for wild dogs in the Selous Game Reserve, Tanzania. Pup mortality (emergence through 1 year) in our region was 24.4±8.0% for females and 22.5±7.3% for males. Yearling mortality was 23.0±7.0% for females and 8.2±7.5% for males, with this rate remaining similar for 2 year old females and increasing for males (females, 22.6±8.0%; males, 23.8±4.8%). However, the incidence of mortality in females aged 3 years (42.9±8.0%) and older (50.0±8.0%) was higher than for counterpart males (27.3±4.8% and 32.0±4.8%, respectively). The latter finding is known to lead to increasing male bias in this species in older age classes [25], [35], [39], [45].

Inbreeding depression was simulated as a reduction in pup survival of inbred individuals according to the general model of Morton et al. [46] (although inbreeding depression may affect other components of reproductive fitness including fecundity and adult survival in addition to pup survival):
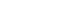
where *S_f_* and *S_0_* are survival rates for individuals with inbreeding coefficients equal to *f* and 0, respectively, and *B* is a constant describing the rate of decline in survival with increasing coefficient of inbreeding. The severity of inbreeding depression is expressed in terms of the number of lethal equivalents per diploid genome in the population of interest (2*B*). We used the nonlinear maximum likelihood approach of Kalinowski & Hedrick [47] to estimate the number of lethal equivalents, using data on inbreeding coefficients derived from studbook records and observed survival rates. From this analysis, we estimate a total of 2*B* = 1.7 lethal equivalents per diploid genome for the KZN wild dog population. We assume that 50% of this genetic load is attributable to lethal alleles, with the remainder composed of detrimental alleles, or those alleles that confer only a probability of dying for an individual that is homozygous for the allele in question. The distribution of the genetic load of a mammal population (i.e., lethal vs. detrimental) is impossible to determine through field observation. The choice of 50% of the genetic load due to lethal alleles is derived from extensive research in *Drosophila* that is routinely applied to analyses across mammals, birds, and other animals of conservation concern [48]. Therefore, given this information, VORTEX reduces the survival probability of an inbred individual during their first year of life by the factor
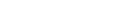
where Pr[Lethals] is the proportion of the total inbreeding effect (number of lethal equivalents) attributable to lethal alleles. The VORTEX model explicitly allows for purging of deleterious alleles over time, with the rate of purging related to the type of genetic load (i.e., a higher proportion of lethal alleles leads to a faster rate of purging).

We initially set the carrying capacity in the model to twice the size of the KZN population as of December 2008 (initial population size = 81; carrying capacity = 162). The carrying capacity was altered in later scenarios to allow exploring its relationship to the probability of extinction in the presence of inbreeding avoidance. We purposely did not include supplementation from human management into the model because of our focus on understanding the future viability of the extant population. Natural immigration from other areas also was not included, because there have been no reports of immigrant wild dogs entering KZN or emigrants successfully reaching populations in other South African provinces.

Although pathogens are known to adversely affect the long-term persistence of African wild dog populations [43], disease frequency and severity is difficult to ascertain due to limited access to dead individuals to determine cause of death and few historical records [49]. Therefore, this variable was excluded from the models because the inclusion of these uncertain data could obscure our focus on the demographic effects of inbreeding avoidance behaviours.

We conducted a sensitivity analysis of our model to specifically identify the demographic and genetic variables that most greatly influenced the growth rates and viability of simulated African wild dog populations. This was accomplished by varying the percentages of females breeding, mortality rates, inbreeding thresholds, carrying capacities, or the percentage of males in the breeding pool by ±25% individually, while keeping all other variables constant. The standard sensitivity index for each variable was calculated as S = [(λ_Base−25%_−λ_Base+25%_)/(0.5* λ_Base_)], where λ was the annual rate of population growth calculated from the simulation, and subscripts *Base*, *Base−25%*, *and Base+25%*, referred to growth rates from models using the baseline parameter value and those increasing or decreasing that parameter value by 25%, respectively [50]. Sensitivity analyses including mortality and percentages of breeding females were completed for each age class separately as well as together to determine whether age-specific characteristics most affected the population. Throughout the sensitivity analyses restrictions were consistently applied on inbreeding by preventing matings that would result in offspring with inbreeding coefficients F>0.20 (*r*>0.40), which would prevent matings only with first-order relatives in the simulation.

To explore the effect on the wild dog population, inbreeding avoidance levels were varied to prevent matings with inbreeding coefficient values (F) greater than 0.20, 0.123 (*r* = 0.246), and 0.063 (*r* = 0.126), which prevent breeding between first-order relatives only, second-order (and more related) kin, and third-order (and more related) kin, respectively. VORTEX determines the suitability of mates by calculating the kinship between individuals based on the pedigree information in the studbook file that is continually updated by the program [41]. Therefore, mating is restricted by familial relatedness and not by allelic similarities that may accumulate over time through genetic drift. These models preventing mating among kin were compared to the baseline scenario that did not include an inbreeding threshold (i.e., one that allowed all relatives to breed). Finally, we examined the influence of carrying capacity on population growth and persistence over time in the presence of inbreeding avoidance behaviours by conducting additional analyses varying this parameter in relation to initial population size.

## Results

### Behavioural Evidence for Inbreeding Avoidance

From 1997 through 2008, we were able to observe 156 situations in which a female had the opportunity to mate with first-order adult kin and 65 situations where unrelated males were available for mating within the pack. While only three inbreeding opportunities resulted in matings, breeding occurred in 72.6% of opportunities to mate with unrelated males, which differed significantly from expected values (*X^2^* = 129.02, df = 1, P<0.001). Opportunities for inbreeding were possible for parents and offspring in the natal pack, parents and offspring after the death of a dominant adult (a reproductive vacancy), and between siblings after dispersal (Table 1). As the population expanded, there was a corresponding increase in the number of opportunities for inbreeding between parents and offspring in natal packs (R^2^ = 0.68, P<0.001) and among siblings (R^2^ = 0.88, P<0.001), but not in number of reproductive vacancies occurring (R^2^ = 0.30, P = 0.06). Most importantly, the frequency of observed incestuous pairings did not rise even while opportunities for inbreeding with close relatives increased (parent-offspring in natal pack: R^2^ = 0.003, P = 0.86; siblings: R^2^ = 0.20, P = 0.15).

**Table 1:** Occasions where inbreeding was possible versus observed in the field within natal packs, after reproductive vacancies, and among siblings in the KZN African wild dog population (adults and yearlings) from 1997 through 2008.

			Parent-offspring in natal pack		Parent-offspring after reproductive vacancy		Siblings after dispersing	
Year	Pop. Size	No. packs	Inbreeding possibilities	Inbreeding observed	Inbreeding possibilities	Inbreeding observed	Inbreeding possibilities	Inbreeding observed
1997	9	2	0	0	0	0	1	0
1998	10	2	2	0	0	0	1	0
1999	15	2	10	0	2	0	1	0
2000	5	1	6	0	0	0	1	0
2001	6	2	0	0	0	0	1	0
2002	14	2	7	0	0	0	2	0
2003	24	3	13	1§	0	0	2	0
2004	31	3	12	0	1	1§	2	0
2005	44	5	17	0	0	0	3	0
2006	59	5	13	0	3	0	3	1†
2007	54	6	12	0	0	0	4	0
2008	64	7	30	0	2	0	5	0
Proportion of cases with inbreeding			1/122 = 0.8%		1/8 = 12.5%		1/26 = 3.8%	

§ Mother-son pair that bred together multiple years.

† Full siblings unfamiliar to each other due to being born 2 years apart and present in the natal pack at different times.

Over the course of the 11-year interval, inbreeding was rarely detected via behavioural observations. In one instance, a full sibling cohort comprised of two males and one female was unknown to each other; these individuals were born into the natal pack at different times and subsequently joined together after dispersal and produced pups. In the second case, a son mated with his mother while in the natal pack, which led to a litter comprised of offspring sired by the son and others sired by the alpha male (his father) [25]. The son in this situation went on to displace his father and fill the reproductive vacancy to continue breeding with his mother (Table 1). The only other inbreeding circumstances were associated with two pairs of third order relatives, one aunt-nephew and one cousin-cousin (or half cousin) coupling, each occurring in different packs. In both of these latter cases, neither of these dogs was familiar with the other, having been raised in different natal packs. Otherwise, opposite sex siblings dispersed and generally formed temporary groups for up to 2 years, but these cohorts never interbred and later joined other groups. Collectively, these observations suggested that African wild dogs were actively recognizing and avoiding breeding with familiar kin.

### Genetic Evidence for Inbreeding Avoidance

**Figure 1:**
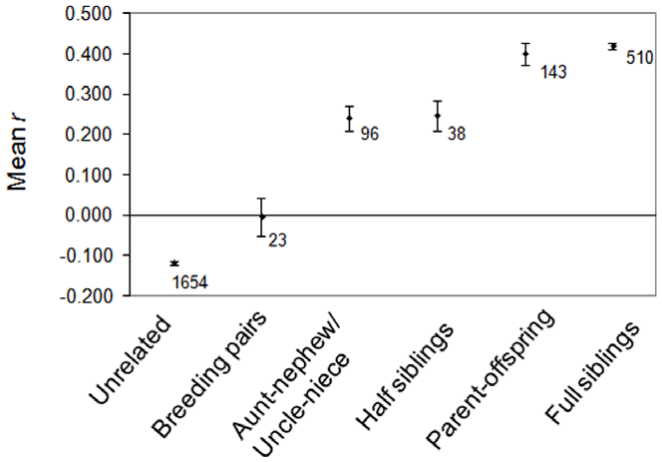
Relatedness comparisons with kinship levels. Mean pairwise relatedness (*r*) for different relationships in the KZN wild dog population with numbers of dyads examined for each category indicated.

Mean pairwise relatedness values calculated for dyads with known relationships were slightly lower than the expected theoretical value of 0.50 for parent-offspring (*r* = 0.40±0.03) and full siblings (*r* = 0.42±0.01), while relatedness values for half siblings were consistent to the theoretical value of 0.25 (*r* = 0.25±0.04; Fig. 1).

To determine whether confirmed breeding pairs of individuals were closely related, we estimated *r* for 23 dyads confirmed via genetic analyses to have produced offspring. Pairwise relatedness of breeding males and females ranged from −0.36 to 0.45 with a mean of −0.04±0.05 (Fig. 1). The latter did not differ from an *r* = 0 (*r* = −0.006±0.004). Of the 23 confirmed breeding pairs, 73.9% (n = 17) were more distantly related than third-order kin in the population. In contrast, there were only two pairs (8.7%) that had *r* values similar to first-order relatives, two pairs (8.7%) with *r* values comparable to second-order kin, and two pairs (8.7%) with relatedness values consistent with third-order relationships. Of the six breeding pairs confirmed via genetic analysis to be related at the third-order kin level or higher, only the mother-son pair was familiar with one another before mating. The other five related pairings were genetic relatives, but consisted of individuals that were never simultaneously in a common natal pack before joining together to breed.

### Population Modelling Analyses

In general, simulated African wild dog populations were influenced most by the (1) proportion of adult females (>2 years) that were able to begin breeding, (2) mortality of females 3 years and older, and (3) inbreeding thresholds that limited the number of suitable mates (Table 2). Other tested factors (e.g., 2 year old female mortality, adult male mortality, carrying capacity, pup mortality, and percentage of males in the breeding pool) were less sensitive to variation in the model (Table 2).

**Table 2:** Sensitivity analyses for selected model input variables with a ±25% variation range in values for a simulated African wild dog population.

Model Parameter	−25%	Baseline	+25%	S
*Females >2 yr first breeding (%)	22.3	29.7	37.1	−0.1523
*3 yr old female mortality (%)	32.2	42.9	53.6	0.0935
*>3 yr old female mortality (%)	37.7	50	62.5	0.0929
*Inbreeding avoidance				
(F threshold)	0.15	0.2	0.25	−0.0795
2 yr old female mortality (%)	17	22.6	28.3	0.0560
Adult male mortality (%)	2 yr = 17.9	2 yr = 23.0	2 yr = 29.8	
	3 yr = 20.5	3 yr = 27.3	3 yr = 34.1	
	>3 yr = 24.0	>3 yr = 32.0	>3 yr = 40.0	0.0358
Carrying capacity (individuals)	122	162	203	−0.0312
Pup mortality (%)	F = 18.3	F = 24.4	F = 30.5	
	M = 16.9	M = 22.5	M = 28.1	0.0303
Males in breeding pool (%)	36	48	60	−0.0093

* Indicates the variables with the highest model sensitivity (S).

**Figure 2:**
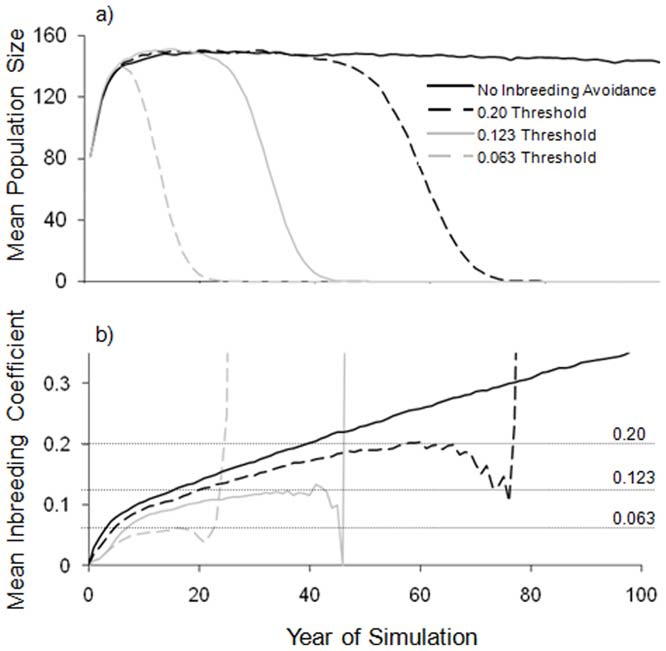
Projections with and without avoidance. Mean projected population size (a) and mean inbreeding coefficients (b) of simulated African wild dog populations over 100 years without inbreeding avoidance behaviours, with prevention of parent-offspring and full-sibling matings (F = 0.20), with prevention of half-sibling matings and higher (F = 0.123), and with prevention of aunt-nephew/uncle-niece matings and higher (F = 0.063). Dotted horizontal lines in (b) indicate inbreeding thresholds. The erratic behavior of mean inbreeding coefficients just before extinction is the result of very small population sizes that lead to unusual mean values near F = 1.0.

**Figure 3:**
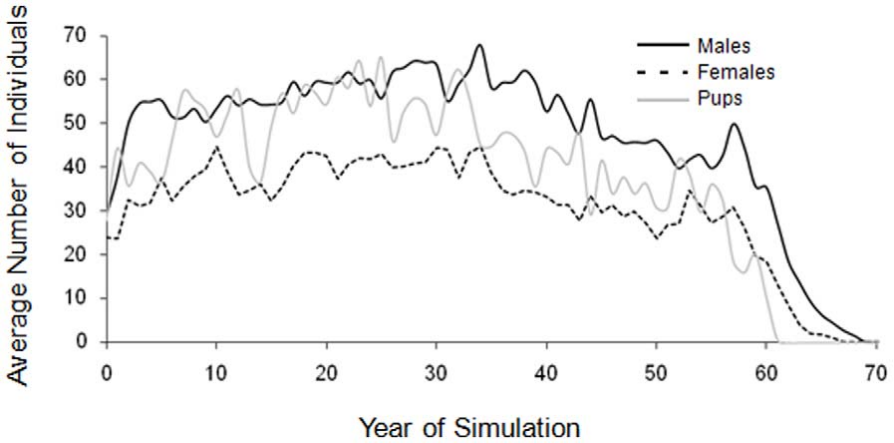
Population composition with avoidance. Average number of adult male, adult female and juvenile wild dogs in simulated populations maintaining an inbreeding threshold of F = 0.20.

In support of the hypothesis that inbreeding avoidance is a significant predictor of population persistence over time, all models that included an inbreeding threshold demonstrated a probability of extinction of 100% within 100 years. The model with mild inbreeding avoidance (to exclude only first-order relative matings) revealed that simulated populations went extinct within 63.1±0.2 years (Fig. 2a). Populations avoiding mating with second-order and more related kin survived 37.0±0.1 years, whereas those that also did not pair with third-order kin became extinct after only 18.7±0.08 years (Fig. 2a). The model not preventing inbreeding had only a 1.6±0.4% chance of extinction before 100 years, and the small percentage of simulations declining to *N* = 0 lasted 50.6±7.4 years. All population models grew rapidly in the first 6 years of the simulation with mean stochastic growth of 13.8±0.01% (no threshold), 15.3±0.02% (F = 0.20), 15.0±0.02% (F = 0.123), and 13.9±0.02% (F = 0.063) until reaching a carrying capacity set at twice the size of the initial population. During years of population expansion, genetic diversity was maintained more effectively in cases that presented the strongest inbreeding avoidance behaviours (Fig. 2b and Table 3). Populations with inbreeding thresholds preventing first-order relative matings (t-test: *t*_10_ = −2.75, P = 0.02), second-order matings (*t*_10_ = −5.58, P<0.001), and third-order matings (*t*_10_ = −7.18, P<0.0001) retained gene diversity better than those without inbreeding avoidance. Despite improved retention of gene diversity in the absence of inbreeding, the limited availability of suitable mates eventually led to demographic failure in these populations. While populations without an inbreeding threshold remained near carrying capacity for the 100 year simulation, in cases where inbreeding was prevented, reproduction slowed, and then pup production stopped due to the presence of only related individuals remaining as potential mates (Fig. 3). Reproduction ceased completely when, for example, first-order relatives were prevented from mating, even when eight females and 12 males remained in the population (Fig. 3). Once inbreeding thresholds began to influence the numbers of individuals that were able to breed, there were dramatic population declines that superseded previous benefits from retained genetic diversity (Fig. 2b and Table 3).

**Table 3:** Average percent gene diversity lost annually before inbreeding thresholds were reached and population growth rates after thresholds began influencing mate availability in simulated African wild dog populations experiencing a range of levels of inbreeding avoidance.

	Loss of gene diversity before inbreeding threshold (%)	Population growth rate after inbreeding threshold (%)
No Inbreeding Avoidance	−0.43	11.57
F = 0.200	−0.39	−5.73
F = 0.123	−0.32	−13.62
F = 0.063	−0.27	−28.65

**Figure 4:**
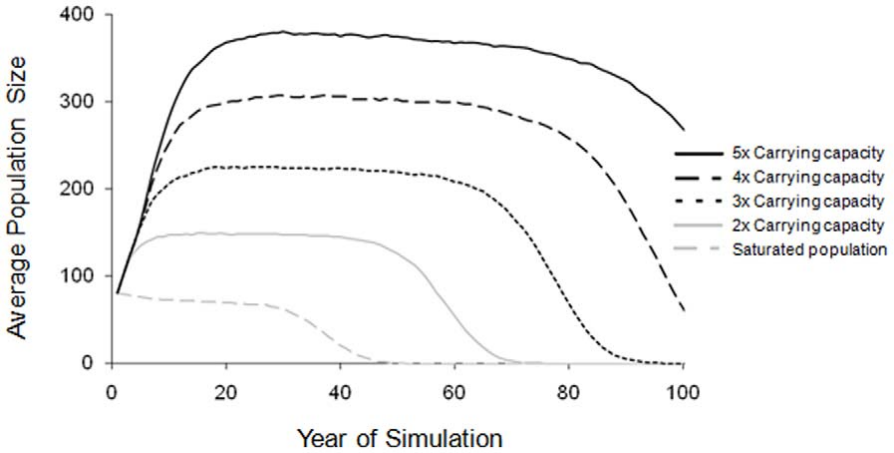
Carrying capacity determines persistence. Average projected size of simulated wild dog populations over 100 years with the carrying capacity parameter set at varying levels in relation to initial population size. Model assumes an inbreeding avoidance threshold of F = 0.20.

Varying the carrying capacity also influenced growth potential and long-term viability of populations (Fig. 4). Simulations with carrying capacities one, two, and three times the initial population size (81, 162, and 243 individuals, respectively) had a 100% chance of extinction before 100 years and survived an average of 40.9±0.2 years, 62.9±0.3 years, and 80.9±0.4 years, respectively. In contrast, models set to carrying capacities of four and five times the initial population (324 and 405 individuals, respectively) were considerably more likely to persist than smaller areas with lower carrying capacity (54.1±1.6% and 17.3±1.2% probabilities of extinction, respectively; Fig. 4).

## Discussion

Results from our integrated behavioural, genetic, and demographic evaluation support our hypothesis that inbreeding avoidance is present in the African wild dog, and suggest that individuals within this species have the capacity to discriminate between kin and non-kin through ‘recognition by association’. These animals most likely learned during rearing to recognize familiar individuals [51].

Our finding that wild dogs had an apparent ability to recognize related kin by association was compatible with our earlier discovery of a higher than expected rate of reproductive sharing in this same population [25]. This previous investigation determined that in packs containing siblings and half siblings of the alpha individuals, subordinate males sired up to 45% of pups, and subordinate females whelped litters in half of all years. Although facilitating the maintenance of genetic diversity in this small, reintroduced population [15], this strategy of shared parentage could, in theory, make it challenging for offspring to distinguish parents from aunts, uncles, and non-relatives. In such circumstances, kin recognition by association would be strongly favoured. Our observations of no inbreeding between siblings, aunts, uncles, nieces, and nephews that shared packs suggested that matings were avoided based on their ability to recognize kin by familiarity to avoid incest. This was also supported by a limited number of cases where interbreeding took place between relatives that had no prior association with each other. Kin recognition based on prior association has also been documented in several other vertebrate species, including the long-tailed tit (*Aegithalos caudatus*) that learns contact calls of close relatives [52] and the naked mole rat (*Heterocephalus glaber*) that differentiates between odours of kin and non-kin [53].

Because of the relative stability and long tenure of African wild dog packs [54], avoidance behaviours based on familiarity would almost always prevent matings of first-order relatives while likely decreasing the chances of mating with second-order relatives. However, some second-order relatives and most cousins would be unfamiliar to each other if originating from different packs. We observed that pairings between unfamiliar kin were avoided less often. Therefore, we suspect that avoidance mechanisms have contributed to maintaining an inbreeding threshold in KZN ranging from F = 0.123 to F = 0.063. This threshold may increase with time as the population becomes more interrelated and more cases of inbreeding occur between third-order relatives that may have been temporally or spatially separated.

Although we determined that wild dogs sustained significantly more genetic variation annually by avoiding incestuous matings, our simulations illustrated the potential adverse consequences on future demographics, especially while populations were declining and becoming more isolated. Specifically, without the supplementation of unrelated individuals through natural immigration or management, modelling demonstrated that the number of suitable, unrelated mates continued to dwindle, and inbreeding thresholds eventually were reached. This, in turn, led to marked negative population growth, rapid population decline, and nearly certain population extinction within 100 years. In fact, there is real-life evidence that inbreeding avoidance may have contributed to the marked decline in this African wild dog population in the 1980s and 1990s, a time when all individuals were descendants from the same pack [26]. Although both reproductively capable males and female wild dogs were present, breeding completely stopped and only recommenced after unrelated individuals were translocated to rebuild the population [27]. Similarly, a population of Scandinavian wolves (*Canis lupus*) was sustained at fewer than 10 individuals within one pack for years followed by exponential growth after the arrival of a single immigrant [55].

Our simulations also were useful for generating new insight into the significance of long-distance dispersal, which already had been recognized as important for maintaining gene diversity in the African wild dog [23]. It is well established that opportunities for offspring to reproduce are only created by leaving close relatives in the natal pack to find mates and form new breeding packs [22], [23]. But emigration also poses significant risks in this species, predominantly mortality that is 1.5 and 1.4 times higher annually for dispersing males and females, respectively, compared to non-dispersing counterparts [39]. This risk also tends to be 1.5 fold greater for females than males due to a longer duration of ‘floating’ between packs [39]. When the corresponding high mortality rates in KZN that include dispersal costs were incorporated into our simulations, the population was particularly sensitive to the loss of adult females, thus creating demographic vulnerability. Others have suggested that emigration exceeding immigration within isolated populations of cooperative breeders can lead to dispersal becoming detrimental, especially as groups decrease below a critical threshold size needed for hunting [56], [57] and caring for young [58]. Consequently, inbreeding avoidance in small, fragmented groups of African wild dogs could drive both dispersal and a mate-finding Allee effect [27], [57] that, in turn, pushes the overall population into a steady decline.

The advantage for males or females to avoid or accept incestuous matings has been reported to depend on the degree of reproductive investment for each sex and the strength of inbreeding depression [18]. Specifically, mutual mate choice for avoiding interbreeding with relatives should evolve when reproductive investment is symmetrical between the sexes and when inbreeding costs are high, thus favouring outbreeding [59]. Supporting this hypothesis, both male and female African wild dogs display high reproductive investment in young within their cooperative breeding system [58], and here we found evidence of inbreeding avoidance through selective mating. This also suggests that the deleterious effects of inbreeding in this species have the potential to be severe. Therefore, wild dogs may have evolved inbreeding avoidance behaviours because the cost of investing in energetically expensive gestation and/or parent care of pups is larger than the fitness payoff of producing more homozygous offspring. Additionally, because higher dispersal risks broaden the conditions favouring inbreeding tolerance [60], the detrimental effects of wild dog relatives interbreeding must be severe to warrant costly dispersal behaviours.

Our modelling indicated that, paradoxically, the same mechanism that evolved to prevent incestuous matings and to maintain genetic diversity could promote population extinction in KZN within 2 to 4 decades simply because too few potential mates are available for dispersing individuals. Other species also have experienced the negative effects of inbreeding avoidance on population demographics, including contributing to extinction prior to the onset of serious inbreeding depression [61]. For example, the red-cockaded woodpecker (*Picoides borealis*), which avoids mating with first-order relatives while having a short dispersal distance, has been found to be highly susceptible to population decline and extinction in the absence of translocations of new individuals [62]. Additionally, offspring of the acorn woodpecker (*Melanerpes formicivorus*) do not fill reproductive vacancies to breed with a parent, which has resulted in population declines of 1.8 to 2.3% annually [63]. Similarly, the African wild dog faces demographic failure because historically important dispersal corridors have divided populations that were previously connected. Strong selection pressures are possibly currently acting against the very same kin recognition and dispersal behaviours that long ago evolved to prevent the negative demographic effects of inbreeding.

### Conservation Implications

Our findings demonstrate that African wild dogs in this growing population avoid incestuous matings. Our simulations suggest that, given adequate resources (habitat, prey) and low persecution, populations should be able to sustain robust genetic diversity. However, given the current dire status of wild dog habitat coupled with high levels of persecution [64] and adult mortality [39], [44], our results also indicate that inbreeding avoidance could further compromise the conservation status of this endangered species. Naturally low population densities and high numbers of human induced deaths may compound Allee effects created by inbreeding avoidance. Our simulations suggest that these effects may well lead to continuous population declines with the potential for extinction of this particular small population in less than 100 years. While our findings may be less relevant to the long-term future of more stable populations, we argue here that inbreeding avoidance is an important factor for considering the conservation management of small and isolated groups of wild dogs. While maintaining and linking prey-filled protected areas is essential for the long term viability of populations, it also appears imperative to continue translocating wild dogs between population isolates to mimic natural immigration and to mitigate this species' mechanisms involving inbreeding avoidance.

## References

[1] Keller LF, Waller DM (2002). Inbreeding effects in wild populations.. Trends Ecol Evol.

[2] Reed DH, Frankham R (2003). The correlation between population fitness and genetic diversity.. Conserv Biol.

[3] Roelke ME, Martenson JS, O'Brien SJ (1993). The consequences of demographic reductions and genetic depletion in the endangered Florida panther.. Curr Biol.

[4] Keller LF (1998). Inbreeding and its fitness effects in an insular population of song sparrows (*Melospiza melodia*).. Evolution.

[5] O'Brien SJ, Everman JF (1988). Interactive influence of infectious disease and genetic diversity in natural populations.. Trends Ecol Evol.

[6] Frankham R, Ballou JD, Briscoe DA (2002). Introduction to conservation genetics.

[7] Brook TM, Mittermeier RA, Mittermeier CG, Da Fonseca GAB, Rylands AB (2002). Habitat loss and extinction in the hotspots of biodiversity.. Conserv Biol.

[8] Pusey A, Wolf M (1996). Inbreeding avoidance in animals.. Trends Ecol Evol.

[9] Hoogland JL (1982). Prairie dogs avoid extreme inbreeding.. Science.

[10] Foerster K, Delhey K, Johnsen A, Lifjeld JT, Kempenaers B (2003). Females increase offspring heterozygosity and fitness through extra-pair matings.. Nature.

[11] Komdeur J, Richardson DS, Burke T (2004). Experimental evidence that kin discrimination in the Seychelles warbler is based on association and not on genetic relatedness.. Proc R Soc Lond B.

[12] Penn DJ, Potts WK (1999). The evolution of mating preferences and major histocompatibility complex genes.. Am Nat.

[13] Mateo JM, Johnston RE (2000). Kin recognition and the ‘armpit effect’: evidence of self-referent phenotype matching.. Proc R Soc Lond B.

[14] Spielman D, Brook BW, Frankham R (2004). Most species are not driven to extinction before genetic factors impact them.. Proc Natl Acad Sci USA.

[15] Miller KA, Nelson NJ, Smith HG, Moore JA (2009). How do reproductive skew and founder group size affect genetic diversity in reintroduced populations?. Mol Ecol.

[16] Williams RN, Rhodes OE, Serfass TL (2000). Assessment of genetic variance among source and reintroduced fisher populations.. J Mammal.

[17] Miller HC, Lambert DM (2004). Genetic drift outweighs balancing selection in shaping post-bottleneck major histocompatibility complex variation in New Zealand robins (*Petroicidae*).. Mol Ecol.

[18] Kokko H, Ots I (2006). When not to avoid inbreeding.. Evolution.

[19] Legendre P, Anderson MJ (1999). Distance-based redundancy analysis: testing multispecies responses in multifactorial ecological experiments.. Ecol Monogr.

[20] Stephens PA, Sutherland WJ, Apollonio M, Festa-Bianchet M, Mainardi D (2000). Vertebrate mating systems, Allee effects and conservation.. Vertebrate mating systems.

[21] Moller AP, Legendre S (2001). Allee effect, sexual selection and demographic stochasticity.. Oikos.

[22] McNutt JW (1996). Sex-biased dispersal in African wild dogs, *Lycaon pictus*.. Anim Behav.

[23] Girman JG, Mills MGL, Geffen E, Wayne RK (1997). A molecular genetic analysis of social structure, dispersal and interpack relationships of the African wild dog (*Lycaon pictus*).. Behav Ecol Sociobiol.

[24] Cooney R, Bennett NG (2000). Inbreeding avoidance and reproductive skew in a cooperative mammal.. Proc R Soc Lond B.

[25] Spiering PA, Somers MJ, Maldonado JE, Wildt DE, Szykman Gunther M (2010). Reproductive sharing and proximate factors mediating cooperative breeding in the African wild dog (*Lycaon pictus*).. Behav Ecol Sociobiol.

[26] Maddock A (1996). Wild dog demography in Hluhluwe-Umfolozi Park, South Africa. Final report of the wild dog photographic study (ZC 53/5).

[27] Somers MJ, Graf JA, Szykman M, Slotow R, Gusset M (2008). Dynamics of a small re-introduced population of endangered wild dogs over 25 years: Allee effects and the implications of sociality for conservation.. Oecologia.

[28] Maddock A (1995). Wild dogs in Hluhluwe-Umfolozi Park.. Reintro News.

[29] Maddock A (1999). Wild dog demography in Hluhluwe-Umfolozi Park, South Africa.. Conserv Biol.

[30] Somers M, Maddock A (1999). Painted dogs of Zululand.. Afr Wildl.

[31] Graf JA, Gusset M, Reid C, Janse van Rensburg S, Slotow R (2006). Evolutionary ecology meets wildlife management: artificial group augmentation in the re-introduction of endangered African wild dogs (*Lycaon pictus*).. Anim Conserv.

[32] Gusset M, Slotow R, Somers MJ (2006). Divided we fail: the importance of social integration for the re-introduction of endangered African wild dogs (*Lycaon pictus*).. J Zool.

[33] Davies-Mostert HT, Mills MGL, Macdonald DW, Hayward MW, Somers MJ (2009). A critical assessment of South Africa's managed metapopulation recovery strategy for African wild dogs.. Reintroduction of top-order predators.

[34] Spiering PA, Szykman Gunther M, Wildt DE, Somers MJ, Maldonado JE (2009). Sampling error in non-invasive genetic analyses of an endangered social carnivore.. Conserv Genet.

[35] Frame LH, Malcolm JR, Frame GW, van Lawick H (1979). Social organization of African wild dogs (*Lycaon pictus*) on the Serengeti Plains, Tanzania, 1967–1978.. Z Tierpsychol.

[36] Marshall TC, Slate J, Kruuk LEB, Pemberton JM (1998). Statistical confidence for likelihood-based paternity inference in natural populations.. Mol Ecol.

[37] Goodnight KF, Queller DC (1999). Computer software for performing likelihood tests of pedigree relationship using genetic markers.. Mol Ecol.

[38] Wright S (1922). Coefficients of inbreeding and relationship.. Am Nat.

[39] Creel S, Creel NM (2002). The African wild dog: Behavior, ecology and conservation.

[40] Lacy RC, Borbat M, Pollak JP (2005). VORTEX: A stochastic simulation of the extinction process. Version 9.50.

[41] Miller PS, Lacy RC (2005). VORTEX: A stochastic simulation of the extinction process. Version 9.50 User's Manual.

[42] McNutt JW, Parker MN, Swarner MJ, Gusset M (2008). Adoption as a conservation tool for endangered African wild dogs (*Lycaon pictus*).. S Afr J Wildl Res.

[43] Vucetich JA, Creel S (1999). Ecological interactions, social organization, and extinction risk in African wild dogs.. Conserv Biol.

[44] Woodroffe R, Ginsberg JR, Woodroffe R, Ginsberg J, Macdonald D (1997). Past and future causes of wild dogs' population decline.. The African wild dog: Status survey and conservation action plan.

[45] Reich A (1981). The behavior and ecology of the African wild dog (*Lycaon pictus*) in the Kruger National Park..

[46] Morton NE, Crow JF, Muller HJ (1956). An estimate of the mutational damage in man from data on consanguineous marriages.. Proc Natl Acad Sci USA.

[47] Kalinowski ST, Hedrick PW (1998). An improved method for estimating inbreeding depression in pedigrees.. Zoo Biol.

[48] Simmons MJ, Crow JF (1977). Mutations affecting fitness in Drosophila populations.. Annu Rev Genet.

[49] Flacke G (2007). An infectious disease and mortality survey in a re-introduced population of African wild dogs (*Lycaon pictus*) and sympatric domestic dogs (*Canis familiaris*) in northern KwaZulu-Natal Province, South Africa..

[50] Heppell SS, Caswell H, Crowder LB (2000). Life histories and elasticity patterns: Perturbation analysis for species with minimal demographic data.. Ecology.

[51] Blaustein AR, Bekoff M, Daniels TJ, Fletcher DJC, Michener CD (1987). Kin recognition in vertebrates (excluding primates): empirical evidence.. Kin recognition in animals.

[52] Sharp SP, McGowan A, Wood MJ, Hatchwell BJ (2005). Learned kin recognition cues in a social bird.. Nature.

[53] Clark FM, Faulkes CG (1999). Kin discrimination and female mate choice in the naked mole-rat *Heterocephalus glaber*.. Proc R Soc Lond B.

[54] deVilliers MS, Richardson PRK, vanJaarsveld AS (2003). Patterns of coalition forming and spatial association in a social carnivore, the African wild dog (*Lycaon pictus*).. J Zool Lond.

[55] Vilà C, Sundqvist A, Flagstad O, Seddon J, Bjornerfeldt S (2003). Rescue of a severely bottlenecked wolf (*Canis lupus*) population by a single immigrant.. Proc R Soc Lond B.

[56] Creel S, Creel NM (1995). Communal hunting and pack size in African wild dogs, *Lycaon pictus*.. Anim Behav.

[57] Courchamp F, Clutton-Brock T, Grenfell B (1999). Multipack dynamics and the Allee effects in the African wild dog, *Lycaon pictus*.. Anim Conserv.

[58] Malcolm JR, Marten K (1982). Natural selection and the communal rearing of pups in African wild dogs (*Lycaon pictus*).. Behav Ecol Sociobiol.

[59] Parker AG (2006). Sexual conflict over mating and fertilization: an overview.. Proc R Soc Lond B.

[60] Waser PM, Austad SN, Keane B (1986). When should animals tolerate inbreeding?. Am Nat.

[61] Koenig WD, Haydock J, Koenig WD, Dickinson JL (2004). Incest and incest avoidance.. Ecology and evolution of cooperatively breeding in birds.

[62] Daniels SJ, Priddy JA, Walters JA, Young AG, Clarke GM (2000). Inbreeding in small populations of red-cockaded woodpeckers.. Genetics, demography and viability of fragmented populations.

[63] Koenig WD, Stanback MT, Haydock J (1999). Demographic consequences of incest avoidance in the cooperatively breeding acorn woodpecker.. Anim Behav.

[64] Woodroffe R, McNutt JW, Mills MGL, Sillero C, Macdonald DW (2004). The African wild dog.. Wild canids: Status Survey and Conservation Action Plan.

